# Varicella-Zoster Reactivation in a Non-immunized Elderly Multiple Sclerosis Patient While on Delayed-Release Dimethyl Fumarate With Grade 2 Lymphopenia and Profoundly Low CD4+ and CD8+ Cell Counts: A Case Report

**DOI:** 10.7759/cureus.22679

**Published:** 2022-02-28

**Authors:** Bob Daripa, Scott Lucchese

**Affiliations:** 1 Medicine, Grant Government Medical College and Sir J.J. (Jamsetjee Jeejebhoy) Group of Hospitals, Mumbai, IND; 2 Internal Medicine, Neurology, Singapore General Hospital, Singapore, SGP; 3 Neurology, University Hospital, Missouri University Health Care, Columbia, USA; 4 Neurology, Headache, University of Arkansas for Medical Sciences, Little Rock, USA; 5 Neurology, Headache, University of Missouri School of Medicine, Columbia, USA

**Keywords:** non-immunized, disease modifying therapies, cd8+ cell count, cd4+ cell count, absolute lymphocyte count, lymphopenia, opportunistic infection, varicella zoster virus, multiple sclerosis, dimethyl fumarate

## Abstract

Increased susceptibility to opportunistic infections (OI) in multiple sclerosis (MS) patients is a real concern amongst neurologists when using disease-modifying therapies (DMTs). DMTs used in modulating or suppressing the immune system for MS management may risk the patient with lymphocytopenia, raising the possibility of OI; however, this lymphopenia may contemplate as a biomarker for drug response, degree of immunomodulation, and drug compliance. The OI could be reactivation of varicella-zoster, progressive multifocal leukoencephalopathy (PML) induced by John Cunningham virus (JC virus), *Pneumocystis jirovecii* infection, cryptococcal meningitis, atypical mycobacteria, and many more.

We present a non-immunized case of varicella-zoster reactivation with dimethyl fumarate (DMF) therapy. Surprisingly, the patient’s lymphocyte count trend during her previous follow-up visits remained in the range of normal to grade 1 lymphopenia but with her current flared-up rash presentation, she had a profoundly low CD8+ and CD4+ cell counts (CD8+ cell count << CD4+ cell counts) despite an absolute lymphocyte (ALC) level far above 500 cells/µl; in fact, it was 13.6% higher when compared to her last quarterly levels. Controlled trials with DMF claimed no serious infection even with a lymphopenia range of 500-800 cells/µl, which is untrue in real clinics and it would be wise and reasonable to follow the lymphocyte subsets along with ALC to prevent potential opportunistic infections.

Recently, comprehensive strategies were evolved to mitigate OI risk for MS patients while on DMTs. These were not only limited to lymphocyte threshold monitoring but extended to address features in terms of screening recommendation, vaccination advice, the need for antibiotic prophylaxis, neuroimaging, laboratory checkups, medication dosing, and behavioral modifications. Our patient was not immunized with zoster vaccine and, unfortunately, DMF has no proper structured guidelines regarding vaccination against OI prevention as other few DMTs have. Our case could suggest that MS patients need proper vaccination guidelines from the Centers for Disease Control and Prevention (CDC) before starting DMF.

## Introduction

One of the most worrisome issues regarding the management of multiple sclerosis (MS) patients in the real-world clinical practice is increased susceptibility to opportunistic/serious infections (OI) such as varicella-zoster reactivation, development of progressive multifocal leukoencephalopathy (PML), *Pneumocystis jirovecii* respiratory infection, herpes simplex virus encephalitis, cryptococcal meningitis, atypical mycobacteria, latent tuberculosis reactivation, and many other respiratory and urinary tract infections while using the disease-modifying therapies (DMTs) [1,2]. Human immunodeficiency virus (HIV) itself is an important archetype as its association with PML is clearly seen when the CD4+ cell counts fall below 200 cells/µl [3]; likewise, CD8+ T cells are key in restraining John Cunningham virus (JC virus) where its decrement raises the possibility of viral CNS infection [1]. The use of DMTs in modulating or suppressing the immune system for MS management may risk the patient with lymphocytopenia raising the possibility of OI, yet this lymphopenia may contemplate as a biomarker for drug response, degree of immunomodulation [1], and drug compliance [4].

Anti-JC virus antibody index and changes on periodic MRI scans have been quite effective for risk identification and monitoring for PML while treating with natalizumab [5]. Other medications have surveillance programs too, to monitor the increased opportunistic infective risk and the appropriate action it demands [4]. The solicitude of reactivation of latent varicella-zoster virus infection in MS patients secondary to immune modulation is genuine [6]. Varicella-zoster is not an uncommon issue in the general medical world [7] and is certainly not usually considered to be excessively daunting. This condition is more of a painful dermal irritation than a serious risk, with the exception of activation potentially affecting sensitive organs such as the eyes. However, to the MS patient treated with immune system modulation who is lymphopenic, this may represent a momentous threat as disseminated varicella-zoster may develop swiftly, not only causing severe pain but also injuring multiple organ systems if not identified and managed early, though its incidence is unknown [6].

There are some OI risks associated with most MS therapeutics. Dimethyl fumarate (DMF), a well-known immunomodulator and a neuroprotective drug [1], is typically thought of as one of the safer MS medications whose serious infection incidence profile is almost similar to placebo-controlled trials [8], but there have been few reports of PML and zoster while treatment on it, though it is considered quite rare [1,6,9]. We present a non-immunized case that developed zoster rash while on delayed-release DMF. The lymphocyte counts remained in the range of grade 1 and grade 2 lymphopenia posing an unexpectedly high fear for opportunistic infection, such as varicella-zoster virus reactivation as seen in our case. The patient's absolute lymphocyte cell count (ALC) was not below 500 cells/µl when her zoster rash flared up; in fact, it was higher compared to three months prior but she did have very low CD8+ and CD4+ cell counts.

## Case presentation

We present a case of varicella-zoster virus reactivation in a 66-year-old right-handed female, being treated for MS with delayed-release oral DMF. The patient was diagnosed with MS about two decades back, after developing optic neuritis and was started on Interferon beta-1b, continued for approximately four years until Interferon beta-1b was switched to Interferon beta-1a due to intolerance issues. After a decade, the patient developed pancytopenia; details about her lymphocytes and subset status are not known here. Her Interferon beta-1a was discontinued and then started delayed-release DMF, which was continued on a maintenance dose of 240 mg capsules BID for the past six years until her current presentation with the zoster rash. The routine blood workup in these last few years showed normal WBC count with occasional mild grade 1 lymphopenia. She also tried several medications for her fatigue during the latter period including modafinil, armodafinil, and methylphenidate. There is no history of immunization with zoster vaccines.

About three months back, the patient reported feeling fatigued with no new symptoms and unremarkable neurological findings on examination. Laboratory values showed lymphopenia with an ALC of 590 cells/µl (grade 2 lymphopenia), but a normal WBC count. She was found to have indeterminate anti-JC Virus antibody titers of 0.21; other blood parameters were within normal range. As the ALC was >500 cells/µl and the patient was doing well with the current medication, hence it was planned to continue DMF and long-acting methylphenidate was added for her fatigue. 

Recently, the patient visited our clinic again for troublesome new symptoms of unilateral continuous dull headache admixed with a burning character. A red maculopapular rash was seen over the left side of her face in the V1 and V2 dermatomal division of cranial nerve five (CN V) with no neurological deficit on examination. She also had a similar rash over the left occipital region. The rash was painful with papules and tender pustules on an erythematous base. The patient's pupils were equal, reactive with normal visual acuity. Other cranial nerves were well intact. Reflexes were normal with no meningeal signs. Laboratory values at this presentation are shown in a tabular form in Table 1 where the T-cell counts were profoundly low. It is noteworthy that repeat ALC is 13.6% higher compared to three months ago. The IgM antibody to the varicella-zoster virus was positive with no IgG value. This was varicella-zoster reactivation; DMF and methylphenidate were immediately stopped and started on oral valacyclovir at a dose of 1 gram three times a day, oral prednisone 60 mg (1mg/kg) a day for the initial few days with gradual tapering, and local lidocaine ointment with non-steroidal anti-inflammatory drugs (NSAIDs) for local dermal burning pain. Ophthalmology consult ruled out herpes zoster ophthalmicus, retinal necrosis, or vasculitis. Follow-up for the next few weeks noted a good clinical response and the blistering rash gradually cleared up. The DMF was restarted after three weeks with a plan to regularly monitor the ALC and its subsets once a month initially. ALC count before restarting DMF was found to be on lower levels of normal. DMF was restarted at 120 mg twice a day. After one week, the dose was escalated to 240 mg BID on a maintenance dose as per dosage guidelines [8].

**Table 1:** Patient’s laboratory values with normal WBC count. ALC is 13.6% higher compared to the ALC report three months prior (590 cells/µl, grade 2 lymphopenia). The CD4+ to CD8+ ratio is 5.2. The anti-JC virus antibody index present in this case was in the indeterminate range of 0.21. The index cut point for PML risk is 0.9 ALC: absolute lymphocyte count; anti-JC Virus: anti-John Cunningham virus; PML: progressive multifocal leukoencephalopathy ALC grade 1 lymphopenia = 800-lower limit of normal/µl, grade 2 lymphopenia = 500-799/µl, grade 3 lymphopenia = 200-499/µl, and grade 4 is <200 cells/µl Anti-JC virus index value of > 0.40 is positive and < 0.20 is negative

Laboratory values:	Patient’s Count	Normal Reference Range
WBC/L	3.5 × 10^9^ (mild low)	4 -11 × 10^9^
ALC/µl	670 ( Grade 2) (low)	1000 - 4800
T3 cells/µl	107 (low)	600-2500
T4 cells/µl (CD4+ or helper T cell lymphocyte)	89 (very low)	500-1200
T8 cells/µl (CD8+ or cytotoxic T cell lymphocyte)	17 (very low)	150-1000

## Discussion

Our concern regarding this case was not just for reactivation of zoster but potentially increased risk of PML, for which varicella-zoster may be a surrogate [1,6]. Although our patient has no MS relapse or development of any new neurological deficit in view of long exposure to DMF associated with grade 2 lymphopenia, anti-JC virus titers were checked and found to be indeterminate. Asymptomatic PML [2] and fumarate-related PML are a real concern as the latter entity has been reported in older patients where the lymphocyte counts were found in the range of 500- 800 cells/µl [1]. As the anti-JC virus titers were unchanged when compared three months prior, we planned to monitor it every six-monthly for opportunistic risk mitigation as done in the case of natalizumab [2]. This patient had neither sensory-motor deficit, nor any ataxia, vision, or speech difficulty; and including the supportive evidence of lower titers of JC virus index and ALC above 500 cells/µl, we did not opt for brain imaging or cerebrospinal fluid analysis at this time.

DMF does have a recommendation from the manufacturer to monitor the ALC count periodically, which assures no incidence of serious infection with lymphocyte counts between 500 - 800 cells/µL [8]. It is advised to withhold therapy if the ALC drops below 500 cells/µl or any serious infection occurs [8]. The latter is the reason why we stopped DMF. The zoster rash flared up in our patient when the ALC count was not below 500 cells/µl; in fact, it was 13.6% higher compared to three months prior but she did have very low CD8+ and CD4+ cell counts, where the cytotoxic T-cell lymphocytes were more lymphopenic compared to helper T cells. A similar subset lymphocytic trend was reported earlier in a disseminated varicella-zoster case report [6] and this raises a contingency of increased susceptibility of such lymphocytic subset trend patients to acquire viral infection. It is noteworthy that grade 2 and more lymphopenia posses a risk of OI [1] and even after ceasing therapy it is observed that ALC reconstitution may take five months or longer [1], although recent evidence deduces that reconstitution may take less than four months to achieve an ALC of more than 800 cells/µl [10].

Zoster reactivation classically becomes more common with age (> 55 yrs), since advancing age reduces lymphopoiesis impairing cell-mediated immunity [4,6,7], with other factors being stress and smoking [4]. The patient had lower normal to grade 1 lymphopenia as her baseline and it might be because of her age admixed with DMF-induced lymphopenia raising plausibility of reduced lymphocytes, which otherwise could represent vigor of immunomodulation implying better disease control [1]. Clinical studies regarding the incidence of serious infections (which do include opportunistic infections) associated with DMF treatment report similar occurrences as in placebo-controlled trials [6]. Intriguingly, few other reports showed an increased risk of infections like generalized varicella-zoster infection, PML, human herpesvirus 8-related Kaposi sarcoma with DMF where the specific lymphocytic subpopulation was low despite normal or with an ALC count of > 500, hence suggesting a strong recommendation to monitor CD4+ and CD8+ cell counts [9]. 

A recent study on 476 subjects of relapsing MS noted DMF-induced lymphocytopenia where the ALC, CD4+, and CD8+ counts dropped by 44%, 42%, and 53%, respectively, from baseline after one year while the mean value after a year were 1200 cells/µl, 610 cells/µl, and 240 cells/µl, respectively. Few patients developed OI including varicella-zoster where no significant association was observed with either ALC, CD4+, CD8+ counts or to their duration of therapy as these OI were noted before, during, and post DMF therapy [11].

In recent times, strategies have been developed to mitigate opportunistic infection risk for MS patients while on DMTs. These are quite comprehensive in terms of screening recommendations, vaccination advice, the need for antibiotic prophylaxis, neuroimaging, laboratory checkups, medication dosing, and behavioral modifications [2]. Our case is not immunized with zoster vaccine and unfortunately, DMF has no proper structured guidelines regarding vaccination against OI prevention as other few DMTs have [2], although recommended monitoring regimes of lymphocyte threshold for individual DMTs are present [4] and perhaps any grade 2 lymphopenia may be reasonable to start monitoring for opportunistic infection [4]. We illustrate a tabular format of lymphocyte count vigilance and potential opportunistic pathogen mitigate plan while on DMTs (Table 2) [11].

**Table 2:** A simplified tabular format of lymphocytopenia grades, vaccination status, any screening advice, and required action plan for potential opportunistic infection while on few known DMTs DMTs: disease-modifying therapies; VZV: varicella-zoster virus; TB: tuberculosis; HIV: human immunodeficiency virus; DMF: dimethyl fumarate; PML: progressive multifocal leukoencephalopathy; JC virus: John Cunningham virus

Disease modifying therapies (DMTs)	Lymphocytopenic grades and required action plan for potential opportunistic infection while on DMTs treatment	Immunization status or any screening need before therapy
Cladribine	Grade 4 lymphopenia (< 200 cells/µl) : plan for anti-herpes prophylaxis	Immunize with VZV vaccine before therapy starts, if not done earlier.
	Grade 3 lymphopenia (< 500 cells/ µl) : monitor for sign symptoms of infections e.g.- zoster	Screen for latent TB, hepatitis B, hepatitis C, HIV
DMF	Grade 2 lymphopenia (500-799): be vigilant for opportunistic infections and monitor blood counts	Grade 1 (800 cells/µl -lower limit of normal) and Grade 2 (500-799 cells/µl): VZV infection has been reported. No guidelines regarding vaccination.
	Grade 3 (<500 cells/µl) : recommendation is to discontinue therapy	Grade 3 (<500 cells/µl): DMF associated PML been reported
		Moderate (500 -799 cells/µl) to severe (<500 cells/µl) lymphopenia for > 6 months triggered PML in one study
Interferons	-	-
Glatiramer acetate	-	-
Teriflunomide	-	-
Fingolimod	For PML risk stratification need frequent MRIs, also evaluate JCV status (although risk is less than natalizumab)	Immunize with VZV vaccine before therapy starts, if not done earlier
		Before starting treatment exclude chronic active infection like VZV, HIV, hepatitis B, hepatitis C, Tuberculosis
Natalizumab	Frequent MRI and JC Virus (anti-JC Virus antibody index) status for PML	-
Ocrelizumab	CD4+ T cells count of < 250 cells/µl should be ruled out before start of therapy	Antiviral prophylaxis can be considered like herpes, *Pneumocystis jirovecii*
		Preventive screen of malignancy
Alemtuzumab	Herpes, urinary, respiratory infection, *Listeria monocytogenes* - no concrete guidelines for these opportunistic infections	-

## Conclusions

It is well known that lymphopenia occurs in the setting of DMTs and it poses a risk factor for developing opportunistic infections in MS patients. The learning point from our case report is that it may be reasonable to follow lymphocyte subsets (T4 and T8 cell counts) along with ALC in the setting of MS on DMF treatment, eventually preventing potential opportunistic infections like varicella-zoster reactivation. Lymphocyte count alone could be distracting as past evidence show that DMF-related varicella-zoster virus reactivation or PML development occurred with normal or mild lymphopenic patients while, on the contrary, controlled trials claim no serious infection from 500-800 cells/µl lymphopenia range, which is not true in real-world clinics.

Our case could suggest that MS patients require proper vaccination guidelines from CDC before starting DMF. To serve the best interests of our MS patients, it would probably be advantageous to discuss the initial symptoms and signs of zoster reactivation with MS patients in the clinics while they are on DMF, as early recognition will allow timely management and prevent further complications. It would also be wise to exclude any latent infection be it viral, bacterial, or fungal origin.
